# Significant Growth by *Rickettsia* Species within Human Macrophage-Like Cells Is a Phenotype Correlated with the Ability to Cause Disease in Mammals

**DOI:** 10.3390/pathogens10020228

**Published:** 2021-02-19

**Authors:** M. Nathan Kristof, Paige E. Allen, Lane D. Yutzy, Brandon Thibodaux, Christopher D. Paddock, Juan J. Martinez

**Affiliations:** 1 Vector Borne Disease Laboratories, Department of Pathobiological Sciences, LSU School of Veterinary Medicine, Baton Rouge, LA 70803, USA; mnathankristof@gmail.com (M.N.K.); Pallen7@lsu.edu (P.E.A.); Lyutzy1@lsu.edu (L.D.Y.); Bthib34@lsu.edu (B.T.); 2 Rickettsial Zoonoses Branch, Division of Vector-Borne Diseases, Centers for Disease Control and Prevention, U.S. Department of Health and Human Services, Atlanta, GA 30329, USA; Cdp9@cdc.gov

**Keywords:** *Rickettsia*, THP-1 cells, pathogenesis, proliferation

## Abstract

*Rickettsia* are significant sources of tick-borne diseases in humans worldwide. In North America, two species in the spotted fever group of *Rickettsia* have been conclusively associated with disease of humans: *Rickettsia rickettsii*, the causative agent of Rocky Mountain spotted fever, and *Rickettsia parkeri*, the cause of *R. parkeri* rickettsiosis. Previous work in our lab demonstrated non-endothelial parasitism by another pathogenic SFG *Rickettsia* species, *Rickettsia conorii*, within THP-1-derived macrophages, and we have hypothesized that this growth characteristic may be an underappreciated aspect of rickettsial pathogenesis in mammalian hosts. In this work, we demonstrated that multiple other recognized human pathogenic species of *Rickettsia*, including *R. rickettsii*, *R. parkeri*, *Rickettsia africae*, and *Rickettsia* *akari* can grow within target endothelial cells as well as within PMA-differentiated THP-1 cells. In contrast, *Rickettsia bellii*, a *Rickettsia* species not associated with disease of humans, and *R. rickettsii* strain Iowa, an avirulent derivative of pathogenic *R. rickettsii*, could invade both cell types but proliferate only within endothelial cells. Further analysis revealed that similar to previous studies on *R. conorii*, other recognized pathogenic *Rickettsia* species could grow within the cytosol of THP-1-derived macrophages and avoided localization with two different markers of lysosomal compartments; LAMP-2 and cathepsin D. *R. bellii*, on the other hand, demonstrated significant co-localization with lysosomal compartments. Collectively, these findings suggest that the ability of pathogenic rickettsial species to establish a niche within macrophage-like cells could be an important factor in their ability to cause disease in mammals. These findings also suggest that analysis of growth within mammalian phagocytic cells may be useful to predict the pathogenic potential of newly isolated and identified *Rickettsia* species.

## 1. Introduction

Rickettsiae are small, Gram-negative obligate intracellular bacteria that are transmitted to humans via hematophagous arthropod vectors. The growing complement of rickettsial genomes has leveraged the creation of four genetically distinct groups, comprising the ancestral group (AG), spotted fever group (SFG), typhus group (TG), and transitional group (TRG) [1,2,3,4]. With the exception of AG rickettsiae, many species of *Rickettsia* have been identified and recognized as pathogens to humans [5,6,7]. Many members of the SFG, TG, and TRG have been documented to cause mild, self-limiting to severe, sometimes fatal disease in humans [8]. Despite high levels of genetic identity within each group, the pathogenicity of various *Rickettsia* species has been shown to be variable [9,10]. For example, *R. conorii*, the causative agent of Mediterranean spotted fever, is highly pathogenic and associated with severe, sometimes fatal clinical presentation while the SFG species *R. montanensis* has not been conclusively associated with disease in humans [11,12,13,14,15,16]. Moreover, virulence has been reported to differ between strains of the same *Rickettsia* species. Various strains of *Rickettsia rickettsii*, the causative agent of Rocky Mountain spotted fever (RMSF), can vary widely in pathogenicity in mammalian hosts, from highly virulent (Sheila Smith) to avirulent (Iowa) [17]. Multiple genomic analyses have attempted to explain differences in pathogenicity among different species of *Rickettsia,* as well as among strains of the same species; however, to date, the genetic and molecular basis for these differences has not been elucidated.

Vascular endothelial cells have been considered to be the target of rickettsial infection and have been the focus of study for *Rickettsia*-host cell interactions. Rickettsiae induce internalization into these cells, quickly escape from an endosomal-like compartment, and replicate within host cell cytosol. In in vitro models of infections, some species can also spread intra- and inter-cellularly via the polymerization of host cell actin, in a process termed “actin-based motility” [18,19,20,21,22,23,24]. In addition to parasitism of endothelial cells both in vivo and in vitro, we [25,26,27,28] and others [6,29,30,31,32,33] have previously demonstrated that pathogenic *Rickettsia* species invade into and replicate within other cell types, including lymphocytes, neutrophils, hepatocytes, monocytes, and macrophages. Work from our group demonstrated that *R. conorii* is able to invade and proliferate not only in endothelial cells but also in PMA-differentiated macrophage-like cells in vitro. *Rickettsia conorii* cells were located within the cytoplasm of both endothelial cells and THP-1-derived macrophages as intact bacteria and avoided localization in lysosomal compartments [34]. In contrast, *R. montanensis* proliferated in non-phagocytic mammalian cells but not in human macrophage-like cells [34]. In addition, *R. montanensis* could invade THP-1-derived macrophages but was unable to replicate, likely due to its inability to escape from LAMP-2- and cathepsin D- positive compartments. These results demonstrate how closely related *Rickettsia* species can differ considerably in their abilities to proliferate within different cell types and suggest that this characteristic could affect its virulence.

In this work, we determined the growth characteristics of several additional species of *Rickettsia* in both endothelial cells and phagocytes in vitro. We utilized quantitative polymerase chain reaction (qPCR) and immunofluorescent microscopy [34] to characterize the growth of five human pathogenic species and one species not associated with disease in mammals, in an endothelial cell line as well as a PMA-differentiated macrophage-like cell line, THP-1. Results from this work suggest that a species’ ability to invade macrophages and subsequently escape the phagolysosome to establish a replicative niche within the cytosol of professional phagocytes possibly predicts virulence potential within a mammalian host.

## 2. Results

### 2.1. Human Pathogenic Rickettsia Species Are Able to Invade and Proliferate Inside Both Mammalian Endothelial Cells and Mammalian Macrophage-Like Cells

**Figure 1:**
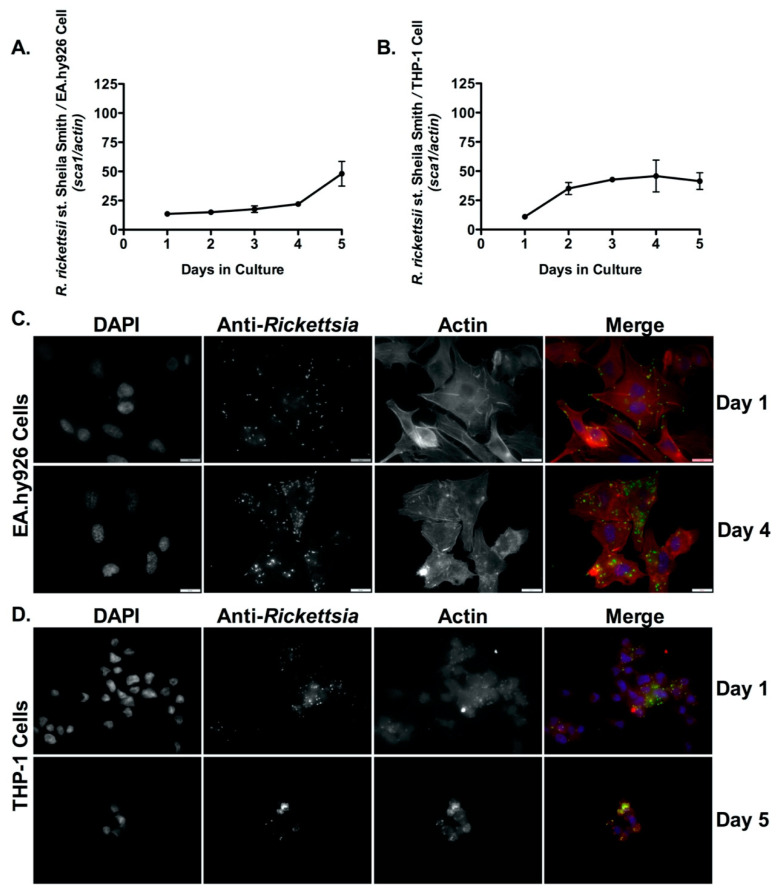
*R. rickettsii* st. Sheila Smith proliferates inside both endothelial cells (EA.hy926) and human derived macrophage cells (THP-1). (**A**,**B**) EA.hy926 cells and PMA-differentiated THP-1 cells were infected with *R. rickettsii* st. Sheila Smith (MOI = 2.5), and genomic DNA was extracted at each time point post-infection. Each time point represents the ratio of *R. rickettsii* st. Sheila Smith *sca1* to the host cell *actin* gene amplified from genomic DNA and determined by quantitative PCR (qPCR). Immunofluorescence microscopy growth analyses in EA.hy926 cells at days 1 and 4 post-infection (**C**,**D**) in PMA-differentiated THP-1 cells at days 1 and 5 post-infection demonstrate significant intracellular proliferation. DAPI (blue) was used to visualize host cell nuclei; anti-*Rickettsia* antibody (RcPFA) followed by Alexa Fluor 488 (green) was utilized to reveal *R. rickettsii* st. Sheila Smith, and Alexa Fluor 546 Phalloidin (red) was used to indicate the host actin cytoskeleton in C and D. Scale bar = 10 μm. A logistic regression test was used to measure significance (*p* < 0.05) in growth over time in both mammalian cell lines in A and B.

Endothelial cell infection by SFG rickettsiae is well documented [34,35,36]. In addition, our laboratory has previously shown that *R. conorii*, the etiologic agent of Mediterranean spotted fever (MSF) is capable of parasitizing non-endothelial cells, including monocyte and macrophages in both in vitro and in vivo models of infection [25,28,34]. We next sought to determine whether other recognized human pathogenic species of *Rickettsia* could grow in phagocytic cells in vitro. To initially test this hypothesis, the endothelial cell line (EA.hy926) and PMA-differentiated THP-1 cells were infected with *R. rickettsii* st. Sheila Smith, the causative agent of Rocky Mountain spotted fever (RMSF) at a MOI of 2.5. At each indicated time point, total genomic DNA was extracted from each sample and analyzed by qPCR to determine the ratio of rickettsial (*sca1*) DNA content to host cell (*actin*) DNA content. As shown in Figure 1A,B, *R. rickettsii* st. Sheila Smith exhibited significant proliferation in both endothelial cells (EA.hy926, *p* = 0.0017) and macrophage-like THP-1 (*p* = 0.0102) cells. A fluorescence microscopy-based growth assay confirmed significant growth within these cell lines (Figure 1C,D).

**Figure 2:**
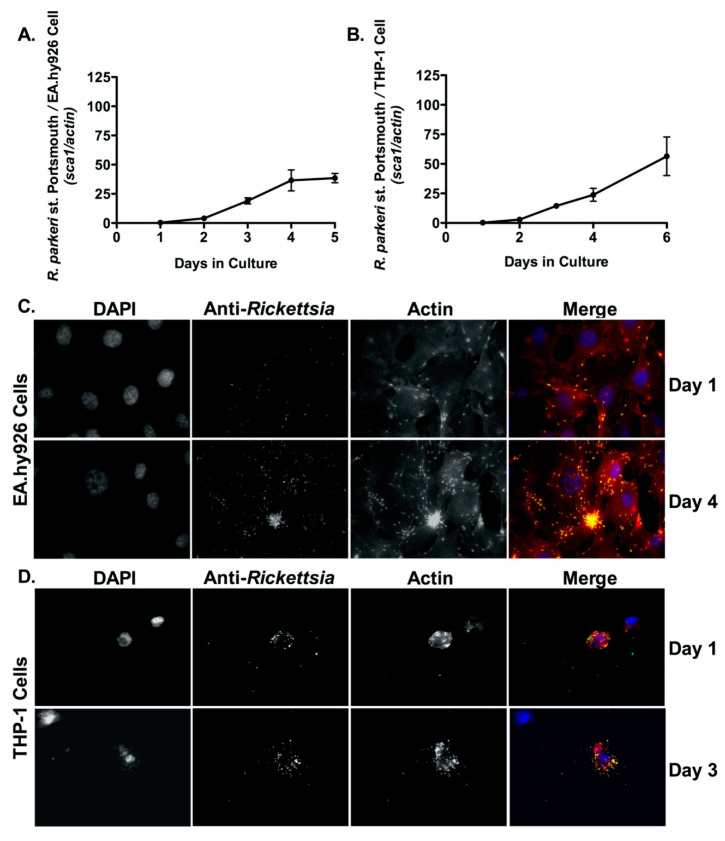
*R. parkeri* proliferates inside both endothelial cells (EA.hy926) and human derived macrophage cells (THP-1). (**A**,**B**) EA.hy926 cells and PMA-differentiated THP-1 cells were infected with *R. parkeri* st. Portsmouth (MOI = 2.5) and genomic DNA was extracted at each time point post-infection. Increase in growth is represented by the ratio of *R. parkeri* st. Portsmouth *sca1* to the host cell *actin* gene determined by quantitative PCR (qPCR). A logistic regression test was used to measure significance (*p* < 0.05) in growth over time in both mammalian cell lines. Immunofluorescence microscopy demonstrated growth in EA.hy926 cells on days 1 and 4 post-infection (**C**) and in PMA-differentiated THP-1 cells on days 1 and 3 post-infection (**D**). Cells were stained with the following antibodies: DAPI (blue) to stain host cell nuclei, anti-*Rickettsia* antibody (RcPFA) followed by Alexa Fluor 488 (green) to stain *R. parkeri* st. Portsmouth, and Phalloidin (red) to stain actin. Scale bar = 10 μm.

*R. parkeri*, a closely related species to *R. rickettsii*, was first isolated in *Amblyomma maculatum* ticks collected from the southern United States more than sixty years ago [37]. However, *R. parkeri* was not established as a human pathogen in North America until 2004 when it was shown that *R. parkeri* was the causative agent of a spotted fever rickettsiosis in a 40-year-old male [37]. We next sought to determine whether this causative agent of spotted fever rickettsiosis exhibited similar growth characteristics in endothelial and macrophage-like cells to *R. rickettsii*. Briefly, EA.hy926 cells and PMA-differentiated THP-1 cells were infected with *R. parkeri* st. Portsmouth at a MOI of 2.5, and growth within these cells was analyzed by qPCR and fluorescent microscopy-based assays as described above. As shown in Figure 2A,B, growth of *R. parkeri* st. Portsmouth is significant in both EA.hy926 cells *(p* < 0.0001) and PMA-differentiated THP-1 cells (*p* < 0.0001) as determined by qPCR. *R. parkeri* growth within these cells was also confirmed by fluorescence microscopy (Figure 2C,D).

We furthered this analysis by determining whether other geographically separated *Rickettsia* species that are confirmed human pathogens would also proliferate in both EA.hy926 and PMA-differentiated THP-1 cells. Mammalian cells were independently infected with *R. akari* (the agent of rickettsialpox) and *R. africae* (the agent of African tick-bite fever) and processed for growth analysis by qPCR and fluorescence microscopy as described above. As shown in Supplemental Figures S1 and S2, each of these species exhibited significant growth in both EA.hy926 cells and differentiated THP-1 cells. These results suggest that growth within phagocytic cells like macrophages may be a common phenotype among *Rickettsia* species that are infectious to mammals.

### 2.2. *R. bellii* and *R. rickettsii* Strain Iowa Are Not Able to Proliferate within Human Macrophage-Like Cells

**Figure 3:**
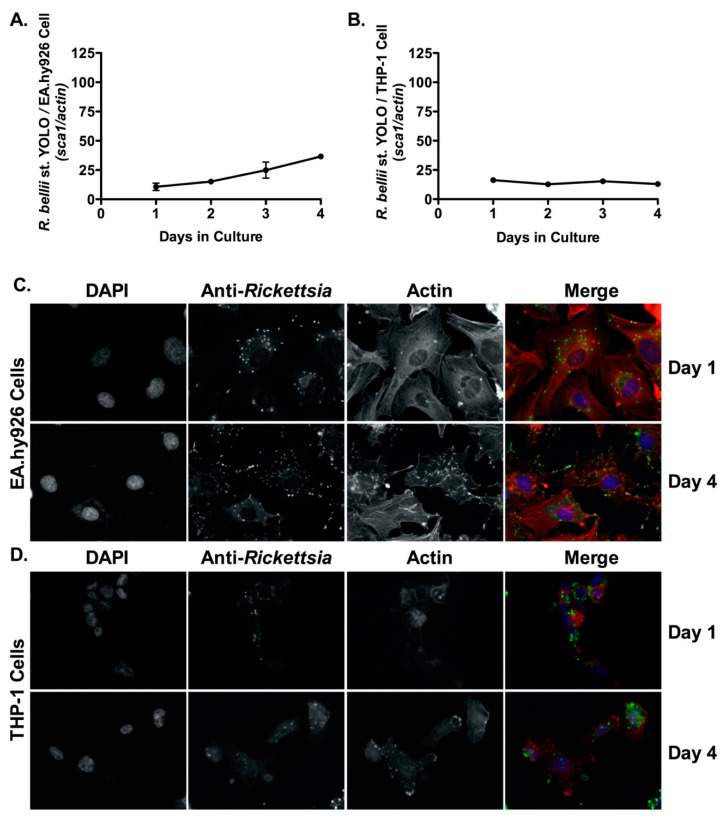
*R. bellii* proliferates inside endothelial cells (EA.hy926) but does not grow in human derived macrophage cells (THP-1). (**A**,**B**) EA.hy926 cells and PMA-differentiated THP-1 cells were infected with *R. bellii* st. Yolo (MOI = 2.5), genomic DNA was extracted at indicated time points and growth was determined by qPCR. A logistic regression test was used to measure significance (*p* < 0.05) in growth over time in both mammalian cell lines. Immunofluorescence microscopy analyses confirmed growth in EA.hy926 cells at days 1 and 4 post-infection (**C**) but not in PMA-differentiated THP-1 cells at days 1 and 4 post-infection (**D**). DAPI (blue) was used to stain host cell nuclei; anti-*Rickettsia* antibody (RcPFA) followed by Alexa Fluor 488 conjugated anti-rabbit IgG (green) was used to stain *R. bellii* st. Yolo, and AlexaFluor 546-Phalloidin (red) was used to stain actin. Scale bar = 10 μm.

*R. bellii* is a *Rickettsia* species that is generally considered to be an endosymbiont of arthropod and insect vectors and is not considered a risk to mammals [38,39,40]. *R. rickettsii* strain Iowa is an avirulent derivative of virulent *R. rickettsii* that was originally isolated through successive passages in embryonated chicken eggs [17]. In comparison to the genome of virulent *R. rickettsii* strain “Sheila Smith”, this strain’s genome contains 492 single nucleotide polymorphisms (SNPs) and 24 deletions that result in several key difference including defective OmpB/Sca5 processing and a truncated OmpA/Sca0 [41]. These differences are thought to contribute to the observed attenuation in virulence within guinea pig models of infection [41]. We next determined whether these two bacteria could invade and proliferate within EA.hy926 cells and THP-1-derived macrophages using qPCR and fluorescence-based microscopy assays. As shown in Figure 3, qPCR and immunofluorescence microscopy analyses demonstrated that while *R. bellii* st. Yolo is capable of growing within EA.hy926 cells; this species did not grow within PMA-differentiated THP-1 cells, similar to what has been previously observed for *R. montanensis* [34]. In addition, *R. rickettsii* strain Iowa grew within EA.hy926 cells but failed to demonstrate significant proliferation within macrophage-like THP-1 cells (Supplemental Figure S3). Collectively, these results demonstrate that the ability to invade into and proliferate within professional phagocytes may be a common trait among recognized, human pathogenic *Rickettsia* species.

### 2.3. Confirmed Human Pathogenic *Rickettsia* Species Evade Localization with Lysosomal Markers within Cultured THP-1-Derived Macrophages

**Figure 4:**
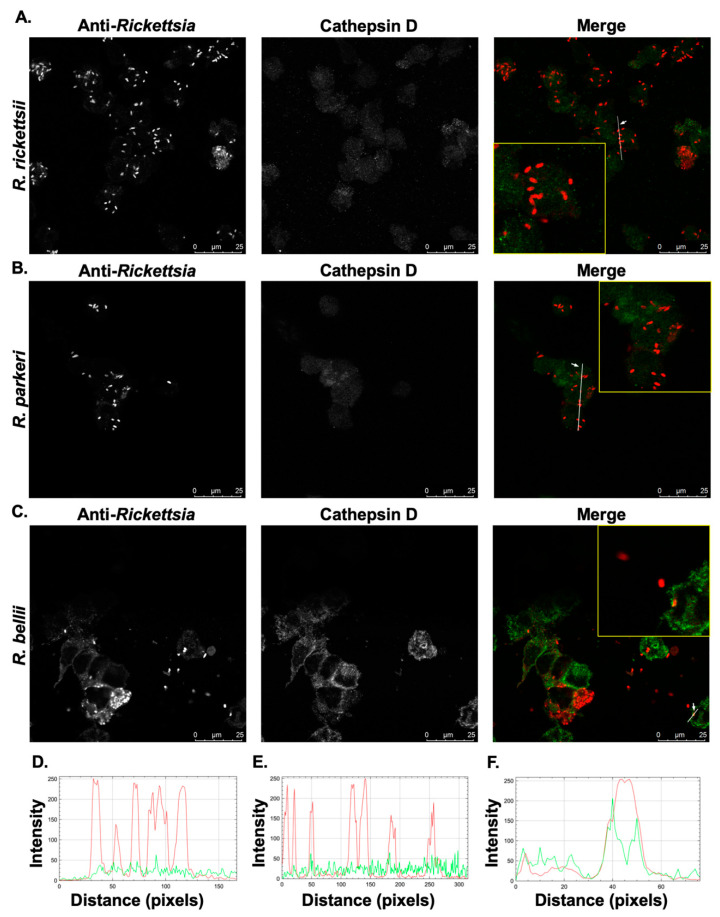
Differences in the co-localization of SFG rickettsial species with the activated, mature form of the lysosomal marker Cathepsin D. PMA-differentiated THP-1 cells were infected with (**A**) *R. rickettsii* Sheila Smith, (**B**) *R. parkeri* Portsmouth, and (**C**) *R. bellii* Yolo at MOIs of 10 for 24 h and then processed for immunofluorescence confocal microscopy analyses. Representative slices from z stacks of infected THP-1-derived macrophage are shown. (**D**–**F**) A generated RGB profile plot documents the relative fluorescence intensity along the indicated white line. Putative co-localization events for *R. rickettsii* (**D**), *R. parkeri* (**E**), and *R. bellii* (**F**) were deemed positive when fluorescence intensities from the green and red channels overlap at a given point in the image. Areas of interest that were used for determination of co-localization are enlarged to show detail (inset). Scale bar = 25 μm.

Our previous work had shown that the growth of *R. conorii* within THP-1 macrophage-like cells is correlated with the ability to evade co-localization with markers of lysosomal compartments, including Cathepsin-D and LAMP-2. On the other hand, *R. montanensis*, that is considered non-pathogenic to humans is rapidly destroyed in compartments resembling lysosomes within PMA-differentiated THP-1 cells [34]. We next determined whether other *Rickettsia* species that significantly grow within THP-1 cells would also evade localization with lysosomal markers, while a species that does not grow fails to avoid destruction within lysosomes. PMA-differentiated THP-1 cells were independently infected with *R. rickettsii*, *R. parkeri*, and *R. bellii* at a MOI of 10 for 24 h and then processed for immunofluorescence microscopy analyses using antibodies against a mature form of Cathepsin D, an aspartic protease that is localized in the lumen of acidified lysosomes. As shown in Figure 4A,B,D,E, *R. rickettsii* and *R. parkeri* both maintain intact intracellular morphology and do not significantly co-localize with Cathepsin-D positive compartments, similar to observations with *R. conorii* [34].

**Figure 5:**
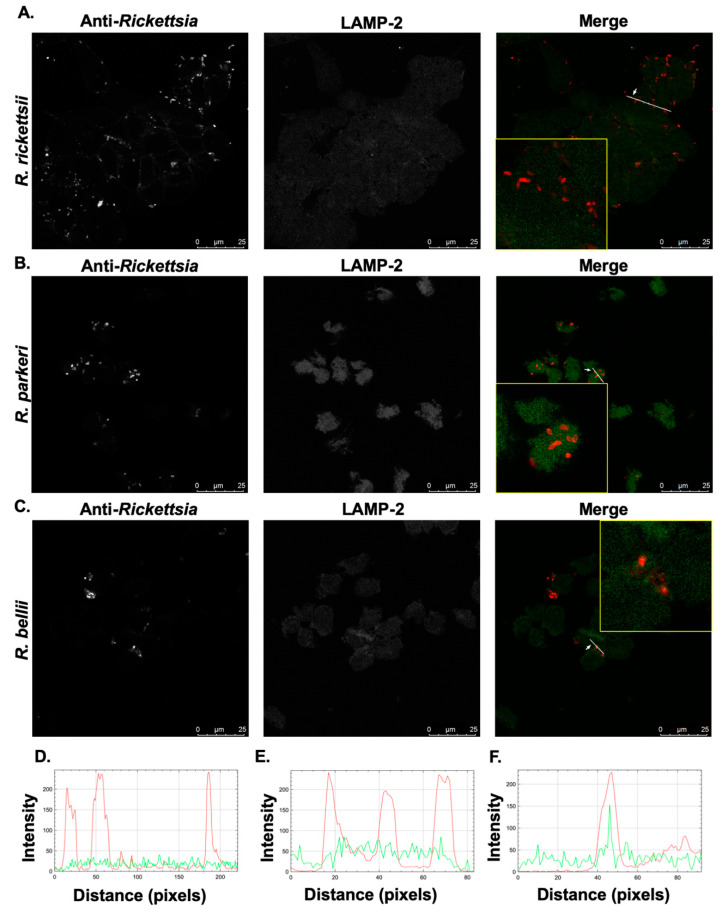
Pathogenic SFG rickettsiae, but not *R. bellii*, avoid co-localization with the lysosomal marker, LAMP-2. PMA-differentiated THP-1 cells were infected with (**A**) *R. rickettsii* Sheila Smith, (**B**) *R. parkeri* Portsmouth, and (**C**) *R. bellii* Yolo at MOIs of 10 for 24 h and then processed for immunofluorescence confocal microscopy analyses. Representative slices from z stacks of infected THP-1-derived macrophage 24 h post-infection are shown. (**D**–**F**) A generated RGB profile plot documents the relative fluorescence intensity along the indicated white line. Co-localization events were deemed positive when fluorescence intensities from the green and red channels overlap at a given point in the image and negative when intensity peaks do not overlap. Areas of interest for *R. rickettsii* (**D**), *R. parkeri* (**E**), and *R. bellii* (**F**) that were used for determination of co-localization are enlarged to show detail (inset). Scale bar = 25 μm.

In contrast, *R. belli* in THP-1 cells occasionally appear fragmented and co-localize with Cathepsin D (Figure 4C,F) similar to what has been previously observed with another, related non-pathogenic SFG *Rickettsia*, *R. montanensis* [34]. These results were further corroborated by a confocal immunofluorescence microscopy analysis using an antibody against LAMP-2 for infected cells at 24 h post-infection (Figure 5).

Taken together, these results demonstrate that genetically related but geographically separated human pathogenic species of *Rickettsia* avoid co-localization with markers of lysosomes and suggest that this phenotype may be a characteristic of recognized and suspected pathogenic SFG rickettsial species.

## 3. Discussion

Previous studies utilizing *R. conorii* and *R. rickettsii* as models for SFG *Rickettsia* pathogens revealed that in addition to parasitism of endothelial cells, pathogenic *Rickettsia* species also infect other non-endothelial cell types including, hepatocytes, lymphocytes, neutrophils, Kupfer cells, adrenal cortical cells, circulating monocytes, and macrophages [25,26,27,28]. Indeed, in a murine model of MSF, intact and presumably replicating *R. conorii* cells were readily detected within peripheral blood monocytes and in tissue resident macrophages at times when the animal is succumbing to fatal disease [28]. Subsequent in vitro studies using a human epithelial cell line, Vero, and PMA-differentiated THP-1 cells demonstrated that a recognized pathogenic SFG species, *R. conorii,* and a “non-pathogenic” SFG species, *R. montanensis*, efficiently grew within epithelial cells; however, only *R. conorii* was able to invade and proliferate inside cultured macrophage-like cells. At 24 h post-infection, *R. montanensis* cells appear fragmented and co-localized with markers of lysosomal compartments, LAMP-2, and Cathepsin D, within PMA-differentiated THP-1 cells [34]. Together, the studies strongly suggested that infection of and subsequent proliferation of SFG rickettsiae within phagocytes may be an underappreciated yet important aspect of pathogenesis.

In the present study, we attempted to determine whether the growth phenotypic difference between *R. conorii* and *R. montanensis* would also be observed in other closely and distantly related *Rickettsia* species. *R. rickettsii*, the causative agent of RMSF, *R. parkeri*, the etiologic agent of a milder spotted fever rickettsiosis, *R. akari*, the agent of rickettsialpox, and *R. africae*, the agent of African tick-bite fever were utilized in these experiments as *bona fide* human pathogenic species. Similar to *R. montanensis*, *R. bellii* has been identified as a tick endosymbiont with no cases of rickettsiosis attributed to *R. bellii* in humans to date [38,42]. *R. rickettsii* strain Iowa is an attenuated variant of *R. rickettsii* and is avirulent in animal models of disease [41]. This strain has been shown to be capable of invading into Vero cells, escaping early phagosomes and undergoing actin-based motility. However, in comparison to the virulent *R. rickettsii* strain “Sheila Smith”, strain Iowa fails to form distinct plaques on Vero cell monolayers [41]. Using a combination of PCR- and fluorescence microscopy-based growth assays [34], we determined similar to *R. montanenesis*, *R. bellii* and *R. rickettsii* strain Iowa significantly proliferated within endothelial cells and failed to grow within macrophage-like cells. In contrast, all of the recognized human pathogenic species tested exhibited significant growth within both endothelial cells and PMA-differentiated THP-1 cells. Furthermore, similar to our previously findings [34], growth of these species within THP-1 cells was correlated with the ability to effectively evade localization with intracellular lysosomal compartments expressing LAMP-2 and activated Cathepsin-D. Together, these results strongly suggest that a phenotypic difference between “pathogenic” and “non-pathogenic” *Rickettsia* species resides in the ability to proliferate within human phagocytic cells. These results also suggest that in the absence of a recognized disease association with a mammalian host, this growth characteristic could be used to predict the potential virulence of newly isolated and identified *Rickettsia* species.

Several studies have documented phenotypic variations with regards to the virulence potential of various *Rickettsia* species and between strains of the same species [34,43,44,45] using in vitro and in vivo models of infection. A few studies have attempted to correlate virulence with the presence or absence of suspected and *bona fide* virulence determinants among recognized human pathogenic *Rickettsia* species and those that have not been determined to cause disease in mammals. For example, a non-virulent variant of *R. rickettsii* (Iowa) contains approximately 492 single nucleotide polymorphisms (SNPs) which, among other changes, result in the inability of the strain to express full-length Sca0/OmpA and to proteolytically cleave Sca5/OmpB [41,44]. A recent study also demonstrated that all pathogenic *R. rickettsii* strains sequenced to date contain an intact gene coding for *Rickettsia* ankyrin repeat protein 2 (RARP-2), a type IV secretion system effector protein that associates with ER-like structures [46]. *R. rickettsii* strain Iowa also contains an internal deletion within *rarp-2* and complementation of this strain with wild-type *rarp-2* fails to restore virulence in a guinea pig model of infection [46]. Interestingly, while RARP-2 is absent in non-pathogenic *R. montanensis* and truncated in *R. bellii*, it is also absent in genomes of several pathogenic strains of *R. prowazekii* and *R. akari* [46], suggesting that the presence or absence of a single gene is not sufficient to determine virulence. Our results also demonstrate that all of the *Rickettsia* species and strains utilized in this study proliferate significantly within the cytoplasm of the endothelial cell line, EA.hy926, strongly suggesting that these bacteria do not have defects in the ability to escape from early intracellular vacuoles during the entry process. Indeed, two gene products, TlyC and Pld, originally studied in typhus group *Rickettsia* species, *R. prowazekii*, have been previously shown to be sufficient to mediate lysis of intracellular vacuoles [47]. Both of these genes are present as intact open reading frames in each of the species utilized in these studies and the predicted encoded proteins exhibit a high degree of amino acid sequence identity compared to each other (Supplementary Figure S4). Therefore, the observed lack of growth by *R. bellii* and *R. rickettsii* strain Iowa in THP-1 cells is likely not due to the absence of these gene products. Because “non-pathogenic” *Rickettsia* species are found within intracellular compartments resembling phagolysosomes, it is possible that secretion of TlyC, Pld, or other membrane lytic enzymes upon entry into phagocytes is somehow perturbed resulting in bacteria that are “trapped” within these bactericidal compartments. Alternatively, pathogenic *Rickettsia* species may possess additional, uncharacterized virulence factors that may lead to the utilization of different internalization pathways within phagocytes, ultimately allowing the bacteria to establish a replicative niche.

Taken together, our results argue that solely using comparative genomic analyses of recognized pathogenic, and “non-pathogenic” *Rickettsia* species may be insufficient to establish virulence potential and that growth characteristics within human phagocytic cells may be a better predictive measure for pathogenicity. The genetic basis and phenotypic mechanisms by which the pathogenic tick-borne rickettsial species are able to elicit diseases in mammals have yet to be fully elucidated and warrant further study.

## 4. Materials and Methods

### 4.1. Cell Lines, Rickettsia Growth, and Purification

EA.hy926 cells were grown in Dulbecco’s modified Eagle’s medium (DMEM; Gibco) containing 10% heat-inactivated fetal bovine serum (Atlanta Biologicals), 1x non-essential amino acids (Corning), and 0.5 mM Sodium Pyruvate (Gibco). THP-1 (ATCC TIB-202™) cells were propagated in RPMI-1640 medium (Gibco) supplemented with 10% heat inactivated fetal bovine serum. Differentiation of THP-1 cells into macrophage-like cells was promoted upon the addition of 100 nM of phorbol 12-myristate 13-acetate (PMA; Fisher). Cells were allowed to differentiate in the presence of PMA for 24 h prior to infection. All cells were allowed to grow at 34 °C in a humidified 5% CO_2_ incubator. *R. africae* st. Eth MA24 [48], *R. rickettsii* st. Sheila Smith [49], *R. rickettsii* strain Iowa [17], *R. parkeri* st. Portsmouth [37], *R. akari* st. Columbia [50], and *R. bellii* st. Yolo [51] were grown in Vero cells and purified as previously described [25,52,53]. All *Rickettsia* species in these studies were used between passage 2 and passage 3 after being received in our laboratories at the LSU School of Veterinary Medicine.

### 4.2. Protein Sequences and Analyses

Protein sequences for a predicted hemolysin (TlyC) and a phospholipase (Pld) from various Rickettsia species were obtained from either the UniProt (https://www.uniprot.org/ (accessed on 20 January 2021)) or NCBI (https://pubmed.ncbi.nlm.nih.gov/ (accessed on 20 January 2021)) online databases. Protein identities were obtained using the Blastp web-based algorithm on the NCBI website using *R. rickettsii* strain Sheila Smith TlyC protein (WP_012151259.1) and Pld protein (WP_012151375.1) as search queries. Indicated “percent identities” from each protein homologue is represented in comparison to the indicated *R. rickettsii* Sheila Smith protein. Protein RefSeq numbers are indicated in parentheses. TlyC homologues: *R. africae* (WP_012719992.1), *R. akari* (WP_012150023.1), *R. bellii* (WP_011477962.1), *R. conorii* Malish 7 (WP_010977712.1), *R. parkeri* (WP_014411035.1), and *R. rickettsii* strain Iowa (WP_012151259.1). Pld homologues: *R. africae* (WP_012720066.1), *R. akari* (WP_012150121.1), *R. bellii* (WP_011476870.1), *R. conorii* (WP_010977832.1), *R. parkeri* (WP_014411111.1), and *R. rickettsii* strain Iowa (WP_012151375.1).

### 4.3. Antibodies

Anti-Rc_PFA_, rabbit polyclonal antibody that recognizes multiple species of SFG rickettsiae, including *R. rickettsii*, *R. conorii*, *R. parkeri*, *R. montanensis*, *R. africae*, *R. akari*, and other species including *R. bellii* and *R. australis* was generated as previously described [19,25]. Alexa Fluor^TM^ 488-conjugated goat anti-rabbit IgG, Alexa Fluor^TM^ 546-conjugated goat anti-rabbit IgG, Alexa Fluor^TM^ 488-conjugated goat anti-mouse IgG, Alexa Fluor^TM^ 546 phalloidin, and DAPI (4′, 6′-diamidino-2-phenylindole) were purchased from Thermo Scientific. Anti-LAMP2 [H4B4] and anti-cathepsin D [CTD19] antibodies were purchased from Abcam.

### 4.4. Analysis of Rickettsial Growth Dynamics

Analysis of rickettsial growth was performed as previously described with slight modifications [34]. Briefly, EA.hy926 cells were seeded into 24-well plates to achieve a working confluency of 2 × 10^5^ cells per well with each time point consisting of 3 infected wells. PMA-differentiated THP-1 cells were seeded in 24-well plates to achieve a working density of 2 × 10^5^–8 × 10^5^ cells per well. Indicated rickettsial species were individually inoculated into mammalian cell lines (EA.hy926 and THP-1) at a multiplicity of infection (MOI) of 2.5. Plates were then centrifuged at 300× *g* for 5 min at room temperature to induce contact between rickettsiae and host cells and then incubated at 34 °C in the presence of 5% CO_2_. Cells were scraped and stored in PBS at −80 °C at each time point post infection until genomic DNA was extracted. Genomic DNA extractions were performed using the PureLink^®^ Genomic DNA Mini Kit (Thermo Fisher Scientific, Austin, Texas 78728, USA) following the manufacturer’s instructions. Growth of individual rickettsial species was determined via a quantitative PCR (qPCR) assay using a LightCycler 480 II (Roche) utilizing iTaq Universal Probes Supermix (Bio Rad) [34] and the following parameters: 10 min at 95 °C; 50 cycles of 95 °C for 10 s, 58 °C for 1 min, and 72 °C for 1 s. This was followed by a cool-down cycle lasting 30 s at 40 °C. Growth was assessed following the amplification of the rickettsial *sca1* gene using the primers sca1-F, sca1-R, and Sca1-Fam and the mammalian *actin* gene was amplified using the primers actin-F, actin-R, and actin-Hex (Supplementary Table S1). The ratio of *sca1* to *actin* was used as the metric to define rickettsial growth per cell as previously described [34]. All experiments were done in triplicate and were performed a minimum of two times.

Immunofluorescence microscopy analysis was also used to verify rickettsial growth within mammalian cells as previously described [34]. Briefly, EA.hy926 and PMA-differentiated THP-1 cells were seeded as described above, onto sterilized glass coverslips. Infections, using the indicated *Rickettsia* species, were performed as described above. Infected monolayers were washed with 1X PBS and subsequently fixed with 4% PFA in PBS for 20 min. Immunofluorescence microscopy analysis was performed essentially as described [34] for all indicated infection time points. *Rickettsia* species within infected cells were visualized with anti-Rc_PFA_ (1:1000), followed by Alexa Fluor^TM^ 488-conjugated goat anti-rabbit IgG (1:1000), followed by DAPI (1:1000) to highlight nuclei and Alexa Fluor^TM^ 546 phalloidin (1:200) to reveal the host actin cytoskeleton. All coverslips were then washed in 1X PBS and then mounted onto glass slides using Mowiol mounting medium. Cells were then viewed and imaged using a LEICA DM 4000 B microscope outfitted with the Nuance FX multispectral imaging system using a final X63 oil immersion optical zoom. Images were processed using Image J software.

### 4.5. Confocal Microscopy Analysis of Lysosomal Compartments

PMA-differentiated THP-1 cells were seeded at 5 × 10^5^ cells per well in 24-well plates on sterile cover slips pre-treated, according to the manufacturer’s protocol, with Poly-L-lysine (Advanced BioMatrix) to promote adherence. The adhered PMA-differentiated THP-1 cells were then infected with the indicated *Rickettsia* species, at a MOI of 10, centrifuged at 300× *g* for 5 min at room temperature to induce adherence, and finally incubated for 24 h at 34 °C and 5% CO_2_. Upon completion of the incubation period, the infected monolayers were washed with 1x PBS and then fixed in 4% paraformaldehyde (PFA) for 20 min prior to staining. After a brief 5-min permeabilization, the infected THP-1 cells were incubated with the primary antibodies anti-Rc_PFA_ (1:1000) and mouse anti-LAMP-2 (1:100) or anti-cathepsin D (1:5500) followed by the secondary antibodies Alexa Fluor 546-conjugated goat anti-rabbit IgG (1:1000) and Alexa Fluor 488-conjugated goat anti-mouse IgG (1:1000). The coverslips were mounted onto slides using Mowiol mounting medium, and images were obtained with the use of a Leica TCS SP8 confocal microscope equipped a with white laser light (WLL) source and with a 100x oil immersion objective kindly provided by the Shared Instruments Facility (SIF) at Louisiana State University. Areas of analysis for co-localization were enlarged by a digital zoom function within the Leica image acquisition software. Subsequent image processing was done with the ImageJ software. Intensity of fluorescence signals and putative co-localization of bacteria with lysosomal markers were analyzed via the ImageJ’s RGB profiler plugin (https://imagej.nih.gov/ij/ (accessed on 20 January 2021)).

## Data Availability

Data sharing not applicable.

## Supplementary Materials

The following are available online at https://www.mdpi.com/2076-0817/10/2/228/s1. Table S1: Primers and probes used for quantitative PCR (qPCR). Figure S1: *R. akari* st. Columbia significantly grows within endothelial cells (EA.hy926) and human derived macrophage cells (THP-1). Figure S2: *R. africae* proliferates within endothelial cells (EA.hy926) and human derived macrophage cells (THP-1). Figure S3: *R. rickettsii* st. Iowa exhibits significant intracellular replication within endothelial cells (EA.hy926) but not human derived macrophage cells (THP-1).
