# Supplementary material for: Significant Growth by Rickettsia Species within Human Macrophage-Like Cells Is a Phenotype Correlated with the Ability to Cause Disease in Mammals
Source: Pathogens. 2021 Feb 19;10(2):228. doi: 10.3390/pathogens10020228 (PMC7934685; doi:10.3390/pathogens10020228)
Supplement: Supplementary file 1 [file pathogens-10-00228-s001.pdf]

Supplementary Table 1. Primers and probes used for quantitative PCR (qPCR).

Rickettsia spp.	Primer/Probe	Sequence (5’ → 3’)
R. africae	Sca1_africae_fwd	CGT GGT ATG TAC GGC ACT AAT AA
	Sca1_africae_rev	TTT CAG CAT CGA ACC CGA TAG
	Sca1_africae	/56-FAM/ACC GGT CAT/ZEN/ATT CTC AAC GCG TCC/3IABkFQ/
R. rickettsii	Rr Sca1 F5271	CAA GCT CGT TAT TAC CCC GAA T
	Sca1_RR_R5371	CTA CCG CTC CTT GGA ATG TTA GAC C
	Sca1_RC_RR_Probe	/56-FAM/TCG GCT TAA/ZEN/GAT ACG GGA AGT/3IABkFQ/
R. parkeri	Rpp Sca-1 (316 bp) FWD	TGA TTC GTA ACA GAT TAG ATG C
	Rpp Sca-1 (316 bp) REV	CCG TAA ATA GAA ACC ACA TGA C
	Rpp Sca-1 PRB Set 2	/56-FAM/ACC GGT CAT/ZEN/ATT CTC AAC GCG TCC/3IABkFQ/
R. akari	Sca1akari_444_fwd	ACT AAC AGA GCA AAC GCC TAA
	Sca1akari_568_rev	CGG TGA TGC CAG AGA AGT ATT
	Sca1_akari(494-518)probe	/56-FAM/CGC CTA CTG/ZEN/TTA GCC CAG CTT CAA/3IABkFQ/
R. bellii	Sca1bellii_13_fwd	GAC AGG GTA GCT GCA GAT ATA AA
	Sca1bellii_162_rev	CCC AAG GAG CTA TGT TCA TTA GT
	Sca1_bellii(57-83)probe	/56-FAM/ TGC AGC GAA/ZEN/AGG CTT AAA CGA TCA AC /3IABkFQ/
**Host Cell Actin**	**Primer/Probe**	**Sequence (5’ → 3’)**
pEC3	Actin-F420	CCT GTA TGC CTC TGG TCG TA
	Actin-R681	CCA TCT CCT GCT CGA AGT CT
	Actin_MS_Probe	/5MAXN/ ACT GTG CCC/ZEN/ATC TAC GAG/3IABkFQ/

All primers and probes for *Rickettsia* species were designed from the rickettsial antigen, Sca1.

Supplemental Figure Legends

Supplemental Figure 1: *R. akari* st. Columbia significantly grows within endothelial cells (EA.hy926) and human derived macrophage cells (THP-1). (A,B) EA.hy926 cells and PMA-differentiated THP-1 cells were infected with *R. akari* st. Columbia (MOI=2.5), and genomic DNA was extracted at each time point post-infection. Each time point represents the ratio of *R. akari sca1* to host cell *actin* genes amplified from genomic DNA and determined by quantitative PCR (qPCR). Immunofluorescence microscopy growth analyses in EA.hy926 cells at days 1 and 3 post-infection **(C)** and in PMA-differentiated THP-1 cells at days 1 and 4 post-infection demonstrate significant intracellular proliferation. DAPI (blue) was used to visualize host cell nuclei, anti-*Rickettsia* antibody (RcPFA) followed by Alexa Fluor 488 (green) was utilized to reveal *R. rickettsii* st. Sheila Smith, and Alexa Fluor 546 Phalloidin (red) was used to indicate the host actin cytoskeleton in **C** and **D**. Scale bar= 10 μm. A logistic regression test was used to measure significance ($p<0.05$) in growth over time in both mammalian cell lines in **A** and **B**.

Supplemental Figure 2: *R. africae* proliferates within endothelial cells (EA.hy926) and human derived macrophage cells (THP-1). (A,B) EA.hy926 cells and PMA-differentiated THP-1 cells were infected with *R. africae* (MOI=2.5), and genomic DNA was extracted at each time point post-infection. Each time point represents the ratio of *R. africae sca1* to host cell *actin* genes amplified from genomic DNA and determined by quantitative PCR (qPCR). A logistic regression test was used to measure significance ($p<0.05$) in growth over time in both mammalian cell lines in **A** and **B**. Immunofluorescence microscopy growth analyses in EA.hy926 cells at days 1 and 5 post-infection **(C)** and in PMA-differentiated THP-1 cells at days 4 and 6 post-infection

demonstrate significant intracellular proliferation. DAPI (blue) was used to visualize host cell nuclei, anti-*Rickettsia* antibody (RcPFA) followed by Alexa Fluor 488 (green) was utilized to reveal *R. africae*, and Alexa Fluor 546 Phalloidin (red) was used to indicate the host actin cytoskeleton in **C** and **D**. Scale bar= 10 μm.

Supplemental Figure 3: *R. rickettsii* strain Iowa exhibits significant intracellular replication within endothelial cells (EA.hy926) but not in human derived macrophage cells (THP-1). (A,B) EA.hy926 cells and PMA-differentiated THP-1 cells were infected with *R. rickettsii* st. Iowa (MOI=2.5), genomic DNA was extracted at each indicated time point post-infection and then growth was determined by qPCR. A logistic regression test was used to measure significance ($p<0.05$) in growth over time and indicated growth in EA.hy926 cells (A), but not in THP-1 cells (B). Immunofluorescence microscopy growth analyses in EA.hy926 cells at days 1 and 4 post-infection **(C)** and in PMA-differentiated THP-1 cells at days 1 and 4 post-infection confirms results from the qPCR analyses. DAPI (blue) was used to visualize host cell nuclei, anti-*Rickettsia* antibody (RcPFA) followed by Alexa Fluor 488 (green) was utilized to reveal *R. rickettsii*, and Alexa Fluor 546 Phalloidin (red) was used to indicate the host actin cytoskeleton in **C** and **D**.

Supplemental Figure 4. TlyC and Pld protein sequence conservation in pathogenic and non-pathogenic *Rickettsia* species. Percent identities of TlyC (**A**) and Pld (**B**) protein homologues were generated from protein sequences (RefSeq) for each indicated *Rickettsia* species when compared to *R. rickettsii* “Sheila Smith” proteins using the NCBI Blastp algorithm.

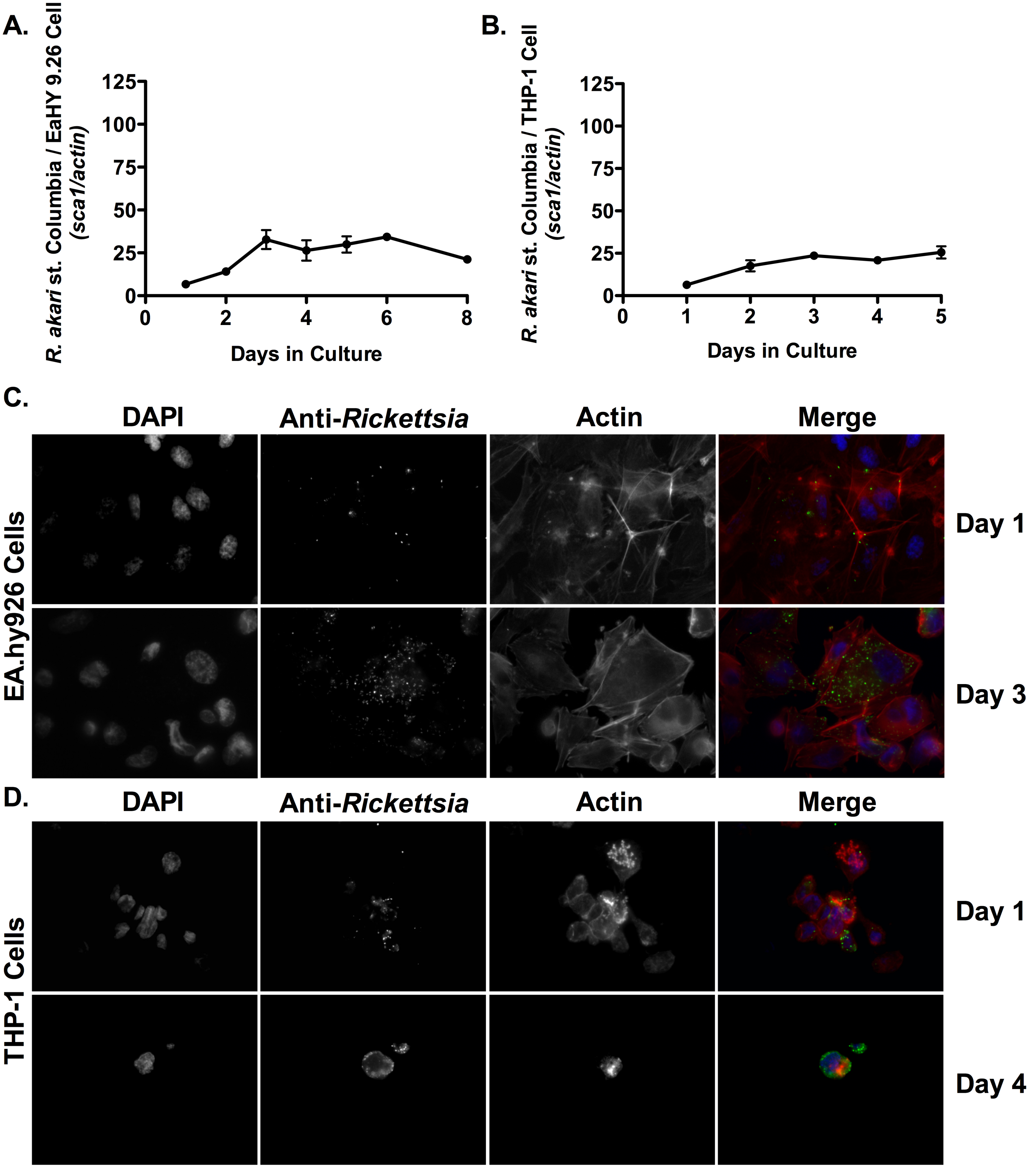

A.
R. akari st. Columbia / EaHY 9.26 Cell
(sca1/actin)
125
100
75
50
25
0
0
2
4
6
8
Days in Culture
B.
R. akari st. Columbia / THP-1 Cell
(sca1/actin)
125
100
75
50
25
0
0
1
2
3
4
5
Days in Culture
C.
DAPI
Anti-Rickettsia
Actin
Merge
EA.hy926 Cells
Day 1
Day 3
D.
DAPI
Anti-Rickettsia
Actin
Merge
THP-1 Cells
Day 1
Day 4

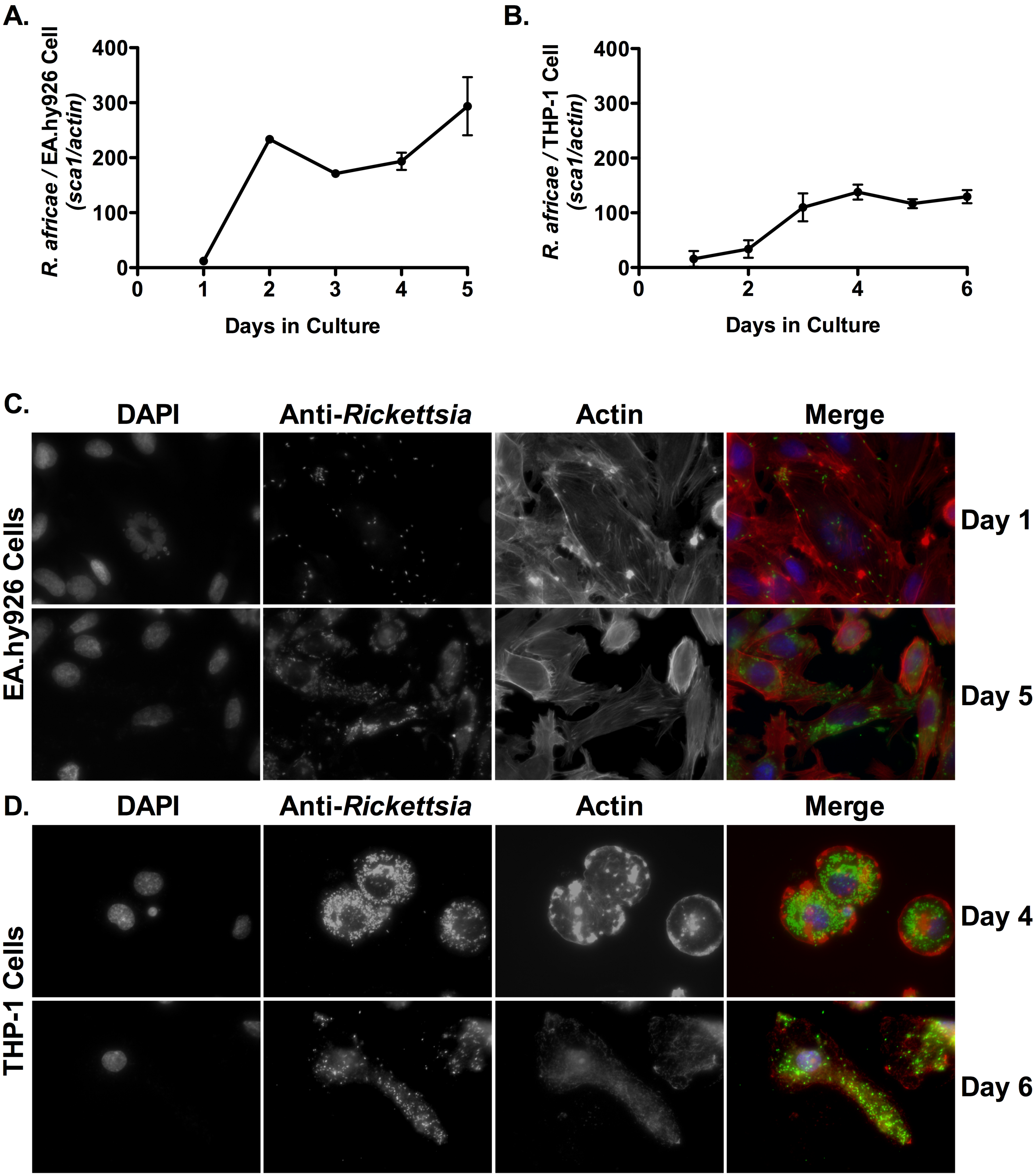
A.
R. africae / EA.hy926 Cell (sca1/actin)
400
300
200
100
0
0 1 2 3 4 5
Days in Culture
B.
R. africae / THP-1 Cell (sca1/actin)
400
300
200
100
0
0 2 4 6
Days in Culture
C.
DAPI
Anti-Rickettsia
Actin
Merge
EA.hy926 Cells
Day 1
Day 5
D.
DAPI
Anti-Rickettsia
Actin
Merge
THP-1 Cells
Day 4
Day 6

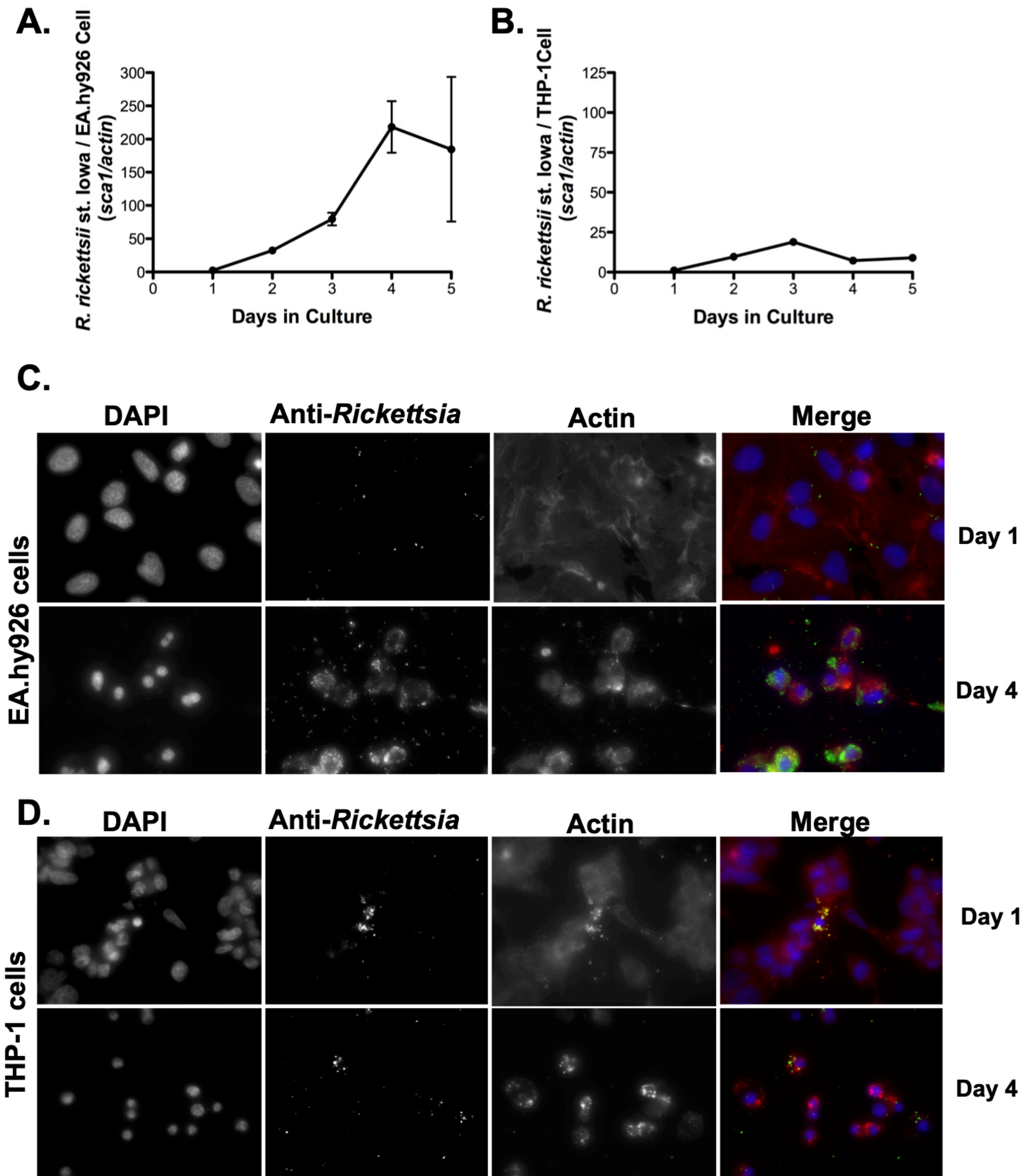
A.
R. rickettsii st. Iowa / EA.hy926 Cell
(sca1/actin)
300
250
200
150
100
50
0
0
1
2
3
4
5
Days in Culture
B.
R. rickettsii st. Iowa / THP-1Cell
(sca1/actin)
125
100
75
50
25
0
0
1
2
3
4
5
Days in Culture
C.
DAPI
Anti-Rickettsia
Actin
Merge
Day 1
Day 4
EA.hy926 cells
D.
DAPI
Anti-Rickettsia
Actin
Merge
Day 1
Day 4
THP-1 cells

A

Species	RefSeq number	Amino acids	% identity
R. rickettsii "Sheila Smith"	WP_012151259.1	299	
R. rickettsii "Iowa"	WP_0121511259.1	299	100
R. conorii	WP_010977712.1	299	99.7
R. africae	WP_012719992.1	299	99.7
R. parkeri	WP_014411035.1	299	99.0
R. akari	WP_012150023.1	301	96.6
R. bellii	WP_011477962.1	301	82.4

B

Species	RefSeq number	Amino acids	% identity
R. rickettsii "Sheila Smith"	WP_012151375.1	200	
R. rickettsii "Iowa"	WP_012151375.1	200	100
R. conorii	WP_010977832.1	200	98
R. parkeri	WP_014411111.1	200	97.5
R. africae	WP_012720066.1	200	96.5
R. akari	WP_012150121.1	200	92
R. bellii	WP_011476870.1	201	79.1
